# Supplementary material for: Sexual harassment before and during the COVID-19 pandemic among adolescent girls and young women (AGYW) in Nairobi, Kenya: a cross-sectional study
Source: BMJ Open. 2022 Oct 17;12(10):e066777. doi: 10.1136/bmjopen-2022-066777 (PMC9577274; doi:10.1136/bmjopen-2022-066777)
Supplement: Author's manuscript [file bmjopen-2022-066777.draft_revisions.pdf]

BMJ Open is committed to open peer review. As part of this commitment we make the peer review history of every article we publish publicly available.

When an article is published we post the peer reviewers' comments and the authors' responses online. We also post the versions of the paper that were used during peer review. These are the versions that the peer review comments apply to.

The versions of the paper that follow are the versions that were submitted during the peer review process. They are not the versions of record or the final published versions. They should not be cited or distributed as the published version of this manuscript.

BMJ Open is an open access journal and the full, final, typeset and author-corrected version of record of the manuscript is available on our site with no access controls, subscription charges or pay-per-view fees (http://bmjopen.bmj.com).

If you have any questions on BMJ Open's open peer review process please email info.bmjopen@bmj.com

BMJ Open

Sexual harassment before and during the COVID-19 pandemic among adolescent girls and young women (AGYW) in Nairobi, Kenya

Journal:	*BMJ Open*
Manuscript ID	bmjopen-2022-066777
Article Type:	Original research
Date Submitted by the Author:	19-Jul-2022
Complete List of Authors:	Bevilacqua, Kristin; Johns Hopkins University Bloomberg School of Public Health, Department of Population, Family and Reproductive Health<br>Williams, A; Johns Hopkins University Bloomberg School of Public Health, Department of Population, Family and Reproductive Health<br>Wood, Shannon; Johns Hopkins University Bloomberg School of Public Health, Department of Population, Family and Reproductive Health; Johns Hopkins University Bloomberg School of Public Health, Department of Population, Family and Reproductive Health<br>Wamue-Ngare, G; Kenyatta University, Department of Sociology, Gender and Development Studies; Kenyatta University, Women's Economic Empowerment Hub<br>Thiongo, Mary; International Centre for Reproductive Health Kenya<br>Gichangi, P; International Centre for Reproductive Health Kenya; Ghent University, Department of Public Health and Primary Care<br>Decker, Michele; Johns Hopkins University Bloomberg School of Public Health, Department of Population, Family and Reproductive Health; Johns Hopkins University Bloomberg School of Public Health, Department of Population, Family and Reproductive Health
Keywords:	COVID-19, PUBLIC HEALTH, EPIDEMIOLOGY

SCHOLARONE™
Manuscripts

I, the Submitting Author has the right to grant and does grant on behalf of all authors of the Work (as defined in the below author licence), an exclusive licence and/or a non-exclusive licence for contributions from authors who are: i) UK Crown employees; ii) where BMJ has agreed a CC-BY licence shall apply, and/or iii) in accordance with the terms applicable for US Federal Government officers or employees acting as part of their official duties; on a worldwide, perpetual, irrevocable, royalty-free basis to BMJ Publishing Group Ltd ("BMJ") its licensees and where the relevant Journal is co-owned by BMJ to the co-owners of the Journal, to publish the Work in this journal and any other BMJ products and to exploit all rights, as set out in our licence.

The Submitting Author accepts and understands that any supply made under these terms is made by BMJ to the Submitting Author unless you are acting as an employee on behalf of your employer or a postgraduate student of an affiliated institution which is paying any applicable article publishing charge ("APC") for Open Access articles. Where the Submitting Author wishes to make the Work available on an Open Access basis (and intends to pay the relevant APC), the terms of reuse of such Open Access shall be governed by a Creative Commons licence – details of these licences and which Creative Commons licence will apply to this Work are set out in our licence referred to above.

Other than as permitted in any relevant BMJ Author's Self Archiving Policies, I confirm this Work has not been accepted for publication elsewhere, is not being considered for publication elsewhere and does not duplicate material already published. I confirm all authors consent to publication of this Work and authorise the granting of this licence.

For peer review only

Sexual harassment before and during the COVID-19 pandemic among adolescent girls and young women (AGYW) in Nairobi, Kenya

Bevilacqua KG[1] kbevila1@jhu.edu; 1-516-297-4305
Williams A[1] awill137@jhu.edu; 1-207-245-0551
Wood SN[1,2] swood@jhu.edu; 1-410-955-0000
Wamue-Ngare G[3,4] wamue.grace@ku.ac.ke; 8710901-19
Thiongo M[5] maryt@icrhk.org; 254 722 208 652
Gichangi P[5,6,7] gichangip2015@gmail.com; 254 722 208 652
Decker MR[1,2*] mdecker@jhu.edu; 1-410-502-2747

[1] Department of Population, Family and Reproductive Health, Johns Hopkins University Bloomberg School of Public Health, Baltimore, Maryland, USA.
[2] Bill & Melinda Gates Institute for Population and Reproductive Health, Department of Population, Family and Reproductive Health, Johns Hopkins University Bloomberg School of Public Health, Baltimore, Maryland USA.
[3] Department of Sociology, Gender and Development Studies, Kenyatta University, Nairobi, Kenya.
[4] Women's Economic Empowerment Hub, Kenyatta University, Nairobi, Kenya.
[5] International Centre for Reproductive Health-Kenya, Nairobi, Kenya.
[6] Technical University of Mombasa, Mombasa, Kenya.
[7] Department of Public Health and Primary Care, Faculty of Medicine and Health Sciences, Ghent University, Belgium

*Corresponding author:
Michele Decker
615 N Wolfe St
Baltimore, MD 21205
mdecker@jh.edu

Word count: 3,292
Tables: 3
Figures: 1
References: 49
Abstract word count: 299/300

For peer review only

Abstract

Objectives: Sexual harassment among adolescent girls and young women (AGYW) is a prevalent, understudied form of gender-based violence (GBV) with negative impacts on health and well-being. The COVID-19 pandemic raised global concern about GBV within homes; less is known about how it affected GBV in public spaces.

Methods: Present analyses utilize cross-sectional data from a cohort of adolescents and young adults residing in Nairobi, Kenya, restricted to female participants. Data was collected August-October 2020 via phone after implementation of COVID-19 restrictions. Prevalence of past-year sexual harassment and harassment relative to COVID-19 restrictions were calculated for overall sample, and by individual, household, and pandemic-related factors. Multivariate negative binomial regression models examine correlates of 1) past-year sexual harassment and 2) increases in sexual harassment relative to COVID-19 restrictions.

Results: Overall, 18.1% of AGYW experienced past-year sexual harassment at 2020 survey. Among this group, 14.6% experienced sexual harassment pre-COVID-19 only, 18.8% after only, and 66.6% at both timepoints. Among the latter group, 34.9% reported more occurrences following COVID-19 restrictions, 20.6% reported less occurrences, and 44.7% reported no change in occurrence. Overall, 42.0% of AGYW experienced an increase in sexual harassment while 58.0% experienced no increase since COVID-19. In adjusted models, past-year sexual harassment was associated with higher educational attainment (aRR: 2.11; 95% CI: 1.27, 3.52) and inability to meet basic financial needs (aRR: 1.67; 95% CI: 1.05, 2.66). Increased sexual harassment since COVID-19 was associated with having full control to leave the home (aRR: 1.69; 95% CI: 1.00, 2.90).

Conclusions: Sexual harassment among AGYW in Nairobi, Kenya was prevalent before and during COVID-19 restrictions. Safety in public spaces remains a highly gendered issue that impacts women's safety and ability to participate in public life. Prevention and support services to address sexual harassment remain an important element in ensuring safe, sustainable public spaces.

Strengths and limitations:

- This is the first study to examine prevalence of sexual harassment in relation to the COVID-19 pandemic among adolescent girls and young women (AGYW) in Nairobi, Kenya.
- We focus on AGYW, a key developmental time-period during which experiences of violence may have long-term consequences for health, well-being, and future risk of GBV.
- We compare prevalence of sexual harassment before and since the COVID-19 pandemic and examine its correlates by time period to better understand the impacts of pandemic-related restrictions on GBV outside of the home.
- Cross-sectional, retrospective data limits our ability to explore causal relationships.
- Use of a single-item outcome measure may have reduced sensitivity, increasing risk of misclassification (i.e., underreporting) of sexual harassment.

For peer review only

BACKGROUND

Global awareness of sexual harassment has risen steadily since 2017, stemming from the global #MeToo, Times Up, and Generation Equality movements. Yet relative to other leading forms of gender-based violence (GBV), sexual harassment remains understudied. Sexual harassment can include staring, cat calling, forced conversation, and/or stalking by either strangers, peers, acquaintances, or authority figures, and often occurs in public spaces, such as on the street or in workplace or educational settings but can also occur in the home and other private spaces.[1] A recent systematic review meta-analysis found wide-ranging prevalence estimates from 0.6%–26.1%, globally,[2] potentially reflecting differences in the conceptualization and measurement of sexual harassment, which occurs across diverse spaces and perpetrators.[3-5] Sexual harassment can serve as a potent reminder of underlying gender imbalances and predatory sexual climates known as coercive sexual environments,[6] which disproportionately burden adolescent girls and young women (AGYM).[5 7 8]

Experiences of sexual harassment during adolescence and young adulthood can have serious consequences for the health and well-being of AGYW. Sexual harassment contributes to increased risk of work and school absenteeism, compromising AGYW's future economic stability.[9 10] For example, harassment experienced on public transportation may compromise AGYW's labor or educational participation and advancement if they are unable to travel safely.[7 11] Experiences of sexual harassment are also associated with a host of negative mental health outcomes, including stress, anxiety, and fear.[10 12 13] Further, sexual harassment among AGYW is associated with elevated risk of future harassment, as well as physical and sexual violence victimization,[14] reflecting embedded social/environmental risks. Despite its consequences, there is a dearth of research aimed at understanding risk factors for sexual harassment among AGYW,[4] and existing research is largely concentrated in high-income countries or among high-income subgroups within low- and middle-income countries (LMICs).[2]

Mirroring gaps in sexual harassment research in LMICs, few studies have explored sexual harassment specifically within Kenya.[15 16] The limited available literature is qualitative and offers useful insight into sexual harassment among AGYW. In one case study at University of Nairobi, women described sexual harassment as a main barrier to feeling comfortable taking certain classes, using university toilets, and accessing certain spaces on campus, such as the library.[15] Another study among high school AGYW in Nairobi's urban settlements highlighted how sexual harassment experienced in transit to and from school often escalated from verbal harassment to unwanted touching, and when men's attention was not reciprocated, it could lead to violence.[17] Though quantitative assessments of sexual harassment among AGYW in Kenya are not currently available, qualitative findings align with experiences of sexual

For peer review only - http://bmjopen.bmj.com/site/about/guidelines.xhtml

harassment among this population in other settings and highlight the gendered risks AGYW face across public contexts such as school, work, and public transportation.[18,19]

The public nature of sexual harassment distinguishes it from other forms of GBV like intimate partner violence, which occur more often in private spaces. The COVID-19 pandemic raised global concern for GBV in *private* spaces due to increased time in the home with potentially abusive partners,[20] however, the potential impacts of changes to AGYW's mobility and time spent *outside of the home* on experiences of GBV are less clear. For example, closures and mobility restrictions due to pandemic policies may decrease sexual harassment risk in some settings like schools but could increase risk of sexual harassment among AGYW traveling in spaces made more isolated by pandemic restrictions. Early evidence of these dynamics has been observed in Kenya; a 2021 UN report on violence against women during the pandemic showed that 55% of women in Kenya reported that COVID-19 made them feel less safe walking alone at night and 81% perceived an increase in sexual harassment since the start of the pandemic.[21] However, to our knowledge, no studies have explored the prevalence of sexual harassment nor risk factors associated with potential COVID-related increases among the priority population of AGYW in Kenya.

The United Nations' Sustainable Development Goal (SDG) 11 calls for the promotion of safe, resilient, and sustainable cities and settlements.[22] Efforts to achieve this goal must employ a gendered perspective to better understand and address the violence and hostility experienced by girls and women in public spaces.[7] To this end, the present study utilizes cross-sectional data from a cohort of youth in Nairobi, Kenya to examine (1) the prevalence of past-year sexual harassment among a sample of AGYW overall, and relative to the implementation of COVID-19 restrictions, (2) factors associated with any past-year sexual harassment, and (3) factors associated with increased sexual harassment relative to COVID-19 restrictions.

For peer review only

METHODS

Study population

The study population is a subsample drawn from the Nairobi Youth Respondent Driven Sampling Survey (YRDSS), an on-going cohort study of adolescents and young adults first recruited pre-pandemic, in 2019. Eligible participants were age 15-24 years, unmarried, and residing in Nairobi for at least one year at the time of enrollment. In August 2019, 1,357 young men and young women were surveyed, 95% of whom (1,293/1,357) consented for recontact. At 12-month follow-up (August-October 2020) 1,217 (94%) were successfully recontacted. Additional sampling and recruitment details can be found elsewhere.[23] Sexual harassment experiences and their timing relative to COVID-19 pandemic restrictions were assessed at the

For peer review only - http://bmjopen.bmj.com/site/about/guidelines.xhtml

2020 survey wave only; the present analytic sample is restricted to all female participants in the 2020 survey (n=612).

Data collection

COVID-19 restrictions were first implemented in Kenya in March, 2020 when President Kenyatta ordered the closing of schools and non-essential workplaces and barred travel from countries with reported cases of COVID-19, among other restrictions.[24] Due to the implementation of COVID-19 restrictions, trained resident enumerators (REs) conducted data collection by phone in either English or Swahili. All data were collected in accordance with best practices for GBV research and RE's received specialized training specific to GBV protections.[25] To ensure privacy and safety for GBV and other sensitive topics during remote data collection, RE's confirmed participant safety and privacy and rescheduled as needed. GBV support services were provided to all participants within a larger list of supports to minimize risk. Participants received 500 KES or $5 USD per survey completed.

Research Ethics Approval

Procedures were approved by the Ethics Review Committee at Kenyatta National Hospital/University of Nairobi (P310/06/2020) and the Institutional Review Boards at Johns Hopkins Bloomberg School of Public Health (IRB 00012952).

Measures

Primary outcomes

Past-year sexual harassment was assessed with a single item adapted from seminal research on coercive sexual environments:[6] "In the past 12-months, have you experienced unwanted sexual attention or harassment such as verbal comments, staring or leering, or unwanted physical contact like groping or grabbing?"; response options included: never, once, a few times, often, or no response. Among AGYW who reported any past-year sexual harassment, participants were asked to report timing relative to the COVID-19 pandemic restrictions (March 2020); "Have experiences of unwanted sexual attention or harassment happened before COVID-19 restrictions only, since COVID-19 restrictions only, or both?" AGYW who reported sexual harassment both before and after COVID-19 restrictions described changes in occurrences; "Have experiences of sexual harassment changed since the COVID-19 restrictions began?" (response options: increased a lot, increased somewhat, no change, decreased somewhat, decreased a lot). To maximize power for analyses, increased sexual harassment since the pandemic, among those who experienced past year sexual harassment, was modeled dichotomously: AGYW were classified as having increased sexual harassment if they first started experiencing sexual harassment after pandemic restrictions

For peer review only - http://bmjopen.bmj.com/site/about/guidelines.xhtml

began OR if they experienced sexual harassment both before and after pandemic restrictions but reported an increase in occurrence since the start of the pandemic. AGYW were classified as no increase in sexual harassment if they experienced sexual harassment only before the pandemic OR if they experienced sexual harassment before and after pandemic restrictions but reported a decrease in occurrence during the pandemic OR if they experienced sexual harassment before and after pandemic restrictions but reported no change in occurrence relative to the pandemic.

Independent variables

Individual, household, and pandemic-related covariates were measured in 2020. At the individual level, demographic assessments including age, educational attainment, current school enrollment status, and marital status were obtained. AGYW's control over the decision to leave the house and over their own earnings was assessed as part of a broader measure on personal agency via 4-point Likert scale, with categories collapsed for analysis (0="less than full control," or 1="full control").[26] Household-level covariates were comprised of subjective household socio-economic status (SES)[27] and household composition (response options: 0= "living alone," 1= "living with parents with or without others," 2= "living with partner with or without others," 3= "living with others"). The pandemic-related covariate focused on inability to meet basic needs since the start of the COVID-19 pandemic, assessed via 4-point Likert scale and dichotomized for analysis (0= "basic financial needs met," or 1= "basic financial needs not met").

Analysis

We first calculated the proportion of AGYW who experienced sexual harassment in the 12 months preceding the survey and the timing of these experiences relative to the COVID-19 restrictions (only before, only after, or both). Among those who reported sexual harassment both before and after pandemic restriction initiation, we characterized changes in occurrences relative to COVID-19 restrictions (i.e., no change, decreased occurrence, or increased occurrence) and overall changes in sexual harassment since COVID-19 restrictions (i.e., increase or no increase). We examined each past-year sexual harassment and overall increased sexual harassment since COVID-19 restrictions by demographics, with differences assessed via design-based F-statistics. Sexual harassment relative to COVID-19 pandemic restrictions among AGYW who experienced past-year sexual harassment (n=118) were visualized using a Sankey diagram (Figure 1).

Based on unadjusted bivariate associations, multivariate negative binomial regression models were constructed to examine correlates of 1) past-year sexual harassment and 2) increases in sexual harassment

For peer review only - http://bmjopen.bmj.com/site/about/guidelines.xhtml

relative to COVID-19 pandemic restrictions, respectively. All analyses were conducted using Stata 15.1 (College Station, TX) with statistical significance set a priori at $p<0.05$; threshold for non-significant trend set at $p<0.10$. Sampling weights accommodate the RDS study design using RDS-II (Volz-Heckathorn) weights, and modest adjustments for post-estimation and loss-to-follow-up. Statistical testing accounts for survey weights and complex survey design.

Patient and Public Involvement

This community-engaged study incorporated community input throughout its inception, implementation, and dissemination. During its inception, key stakeholders from community-based, youth-serving organizations consulted on study recruitment, survey design, and data collection procedures. Trained RE's were recruited from study communities and provided integral input on survey design and data collection. Findings have been disseminated among key stakeholders, including government representatives, community and faith leaders, community-based organizations, and youth leaders.

RESULTS

Overall, 18.1% of AGYW reported past-year sexual harassment (Table 1). Among those who experienced past-year sexual harassment, 66.6% experienced harassment both before and after COVID-19 restrictions (March 2020), 14.6% experienced harassment only before and 18.8% experienced harassment only after pandemic restrictions. Among those with sexual harassment both before and after COVID-19 restrictions, 44.7% experienced no change in occurrence, 34.9% experienced an increase in occurrence and 20.5% experienced a decrease in occurrence. Among AGWY who reported any past-year sexual harassment, 42.0% experienced an overall increase relative to COVID-19 (i.e., new or increased sexual harassment since March 2020) (Figure 1).

For peer review only

Table 1: Sexual harassment prevalence, timing and changes relative to COVID-19 pandemic restrictions among AGYW, 2020 (n=612)

	weighted % (n)
Experienced in past 12 months	18.1 (118)
Timing relative to COVID-19 restrictions; among those who experienced past-year sexual harassment (n=118)	
Pre-COVID-19 only	14.6 (11)
Post- COVID-19 only	18.8 (20)
Both time periods	66.6 (87)
Changes in occurrence since COVID-19 restrictions; among those who experienced sexual harassment both prior to and during COVID-19 restrictions (n=87)	
No change	44.7 (41)

For peer review only - http://bmjopen.bmj.com/site/about/guidelines.xhtml

Decreased occurrence	20.5 (17)
Increased occurrence	34.9 (29)
Overall increase in sexual harassment since COVID-19 restrictions began, among those who experienced past-year sexual harassment+ (n=118)	
Increase	42.0 (49)
No increase	58.0 (69)

+new or increased sexual harassment since March 2020

Correlates of Past-year Sexual Harassment

In bivariate analyses, past-year sexual harassment was more prevalent among AGYW with greater educational attainment (college/university vs. secondary or lower; 38.5% vs. 15.9%; p<0.01) (Table 2), whereas not being enrolled in school at the time of the survey was marginally associated with past-year sexual harassment (20.9% vs. 14.2%; p=0.11). AGYW who were unable to meet their basic financial needs since COVID-19 were more likely to experience past-year sexual harassment than those able to meet their basic financial needs (22.6% vs. 12.9%; p=0.02). In adjusted multivariate negative binomial models, inability to meet basic financial needs remained significantly associated with past-year sexual harassment (aRR: 1.67, 95% CI: 1.05, 2.66), as did higher educational attainment (aRR: 2.11, 95% CI: 1.27, 3.52) (Table 2).

Table 2: Factors associated with past-year sexual harassment among AGYW, weighted

	Sample (n=612)	Past-year sexual harassment		
	col%	row%	p-value+	aRR (95% CI)++
**Overall**	--	18.1	--	--
**Age group**			0.68	
16-20 years	42.6	19.1		--
20-26 years	57.4	17.3		--
**Highest level of education completed**			<0.01**	
Secondary or lower	90.4	15.9		ref
College / University	9.6	38.5		2.11** (1.27, 3.52)
**Currently in school**			0.11	
No	58.2	20.9		ref
Yes	41.8	14.2		0.82 (0.48, 1.40)
**Marital status**			0.46	
Unmarried	90.2	17.6		--
Married	9.8	22.6		--
**Personal control over decision to leave house**			0.85	
Less than full control	60.0	18.5		--
Full control	40.0	17.6		--
**Control over own earnings**			0.29	
Less than full control	71.7	16.7		--
Full control	28.3	21.7		--
**Relative household SES tertile**			0.41	
Lowest	37.0	14.4		--

For peer review only - http://bmjopen.bmj.com/site/about/guidelines.xhtml

Middle	22.2	19.3		--
Highest	40.7	20.8		--
**Household composition**			0.17	
Live alone	6.3	20.0		--
Lives with parent(s), with or without other(s)	66.4	17.2		--
Lives with partner with or without other(s), excluding parent(s)	10.0	30.7		--
Live with others	17.2	13.4		--
**Ability to meet basic financial needs since Covid-19**			0.02*	
Basic financial needs met	46.6	12.9		ref
Basic financial needs unmet	53.4	22.6		1.67* (1.05, 2.66)

$^{+}$P-value for the design-based F statistic bivariate testing
$^{++}$aRR (adjusted risk ratio) generated through multivariate negative binomial regression with past-year sexual harassment as the dependent variable, among full sample (n=612); all variables listed included in specification accounting for robust standard error clustering by node; weighted
*** $p<0.001$, ** $p<0.01$, * $p<0.05$

Correlates of Overall Increase in Sexual Harassment Since COVID-19

Among AGYW who experienced any past-year sexual harassment, in bivariate analyses, those currently enrolled in school were more likely to experience increased sexual harassment since the start of the COVID-19 restrictions compared to those not currently enrolled in school (59.0% vs. 33.7%; p=0.05). Having full control over one's decision to leave the home was marginally bivariately associated with increased sexual harassment (57.4% vs. 32.2%; p=0.06) (Table 3). In adjusted multivariate negative binomial models, full control to leave the home was associated with overall increased sexual harassment since the start of COVID-19 restrictions (aRR: 1.69; 95% CI: 1.00,2.90) (Table 3).

Table 3: Factors associated with increased sexual harassment since COVID-19 restrictions, among AGYW with past-year sexual harassment, weighted

	Sample (n=118)	Increased sexual harassment since Covid		
	col%	row%	p-value^{+}	aRR (95% CI)$^{++}$
**Overall**	--	42.0	--	--
**Age group**			0.38	
16-20 years	45.0	48.3		--
20-26 years	55.0	36.8		--
**Highest level of education completed**			0.60	
Secondary or lower	79.5	40.4		--
College / University	20.5	48.1		--
**Currently in school**			0.05*	
No	67.3	33.7		ref
Yes	32.7	59.0		1.66 (0.98, 2.80)
**Marital status**			0.66	
Unmarried	87.8	43.0		--
Married	12.2	35.1		--
**Personal control over decision to leave house**			0.06	
Less than full control	61.2	32.2		ref
Full control	38.8	57.4		1.69* (1.00, 2.90)

**Control over own earnings**			0.26	
Less than full control	66.2	46.5		--
Full control	33.8	33.2		--
**Relative household SES tertile**			0.97	
Lowest	29.6	43.3		--
Middle	23.6	43.3		--
Highest	46.8	40.5		--
**Household composition**			0.17	
Live alone	7.0	16.6		--
Lives with parent(s), with or without other(s)	63.3	44.4		--
Lives with partner with or without other(s), excluding parent(s)	17.0	25.9		--
Live with others	12.8	65.1		--
**Ability to meet basic financial needs since Covid-19**			0.59	
Basic financial needs met	33.3	37.2		--
Basic financial needs unmet	66.7	44.4		--

+P-value for the design-based F statistic bivariate testing
++aRR (adjusted risk ratio) generated through multivariate negative binomial regression with increased sexual harassment since COVID-19 restrictions as the dependent variable, among those who experienced past-year sexual harassment (n=118); all variables listed included in specification accounting for robust standard error clustering by node; weighted
*** p<0.001, ** p<0.01, * p<0.05

DISCUSSION

Sexual harassment was pervasive among AGYW in our sample, with 18.1% reporting experiences of sexual harassment in the past year. Among AGYW experiencing past-year sexual harassment, the majority reported those experiences occurring both before and after the start of COVID-19 pandemic restrictions. Within the subset of AGYW who experienced sexual harassment before and after COVID-19 restrictions were implemented, most (44.7%) experienced no change in occurrence of sexual harassment after the start of the pandemic. Overall, 42.0% experienced an increase in sexual harassment since the start of COVID-19 pandemic restrictions. The high prevalence of sexual harassment prior to the start of the pandemic and sustained or increased occurrence of such violence echoes the burdens of other forms of GBV in Kenya[28-31] and underscores the need for attention to sexual harassment and its impacts on health and well-being among AGYW in this setting.

Past-year sexual harassment was associated with AGYW's inability to meet their basic financial needs. These results support a growing body of research that has found lower socioeconomic status to be associated with increased risk of sexual harassment during adolescence.[32-35] Interestingly, past-year sexual harassment was also associated with greater educational attainment, perhaps due to sexual harassment experienced in the university[36] or workplace settings.[37] We note that AGYW who were *not currently* enrolled in school during the COVID-19 pandemic were marginally significantly more likely to report past-year sexual harassment. These results suggest that young women who left school, potentially due to the pandemic, and

For peer review only

more educated women who have already entered the workforce may have heightened risk. Results reflect the ways in which both *lower* socioeconomic status and *higher* educational attainment may contribute to increased time in public spaces, either out of financial necessity or workplace opportunities afforded by an advanced degree and suggest that greater access to educational and economic resources may not be protective against sexual harassment. Further, the increased risk associated with school enrollment suggests that initiative to keep AGYW in school may not protect them from sexual harassment and that further intervention to reduce perpetration in school settings is urgently needed. Adolescence and young adulthood mark pivotal developmental junctures during which educational and economic opportunities shape future health and well-being. While public spaces remain unsafe for AGYW, their ability to participate in education or work will continue to be compromised, compounding their socioeconomic vulnerability and risk of future violence.

A growing body of literature has documented the ways in which pandemic-related restrictions and psychosocial stressors have contributed to increased GBV risk globally.[20 28] In contrast to more private forms of GBV like IPV, sexual harassment is often perpetrated in public spaces, making the potential impact of COVID-related restrictions and stressors less clear. The majority of AGYW who had ever experienced sexual harassment did so both before and after the start of the pandemic, suggesting that for most AGYW, pandemic-related restrictions had minimal impact on their risk of sexual harassment, perhaps reflecting economic realities that prevented many Kenyans from remaining home despite stay-at-home orders.[38] In adjusted models, personal control to leave the home was significantly associated with overall increased sexual harassment since the start of the pandemic among AGYW, and current school enrollment was marginally associated with increased sexual harassment. These results underscore the ways in which mobility restrictions may have presented a double edged sword for AGYW, whereby remaining at home may have placed them at greater risk of IPV or violence perpetrated by family members,[28 39-41] and leaving the home may have made them more vulnerable to sexual harassment in public spaces like work and transit.

Unfortunately, the current policy landscape does not reflect the realities of AGYW at risk for sexual harassment. The Sexual Offenses Act, passed by the Government of Kenya in 2006, defines sexual harassment as persistent sexual advances or requests made by *any person in a position of authority or holding public office*.[42] The law's narrow definition fails to address harassment perpetrated by peers or coworkers, in informal work settings, or in public. Notably, penal code 144(3), part of the Act which prohibited insult to women's modesty or utterances of words or sounds and gestures that, "intrude upon the privacy of the woman or girl,"[42] but fell short of labeling such behavior as sexual harassment, has since been repealed.[43] While current policies fall short, public advocacy efforts and attention surrounding sexual

For peer review only - http://bmjopen.bmj.com/site/about/guidelines.xhtml

harassment are mounting for example, when a young woman was harassed and assaulted by boda boda (motorcycle) drivers in March 2022, large public protests erupted.[44] Increased research is urgently needed to inform policy and intervention to address the unique aspects of sexual harassment experienced during adolescence and young adulthood, including age-specific risk factors for perpetration and victimization as well as health impacts.

Results must be understood in the context of the study's limitations. Cross-sectional, retrospective data was obtained at a single point in time and limits our ability to explore causal relationships. Surveys were conducted via mobile phone, which may have resulted in a study sample of more well-resourced youth; however, post-estimation weights improve generalizability to the overall youth population in Nairobi. To minimize participant fatigue, a single item assessed sexual harassment, which may be less sensitive,[45] increasing risk of misclassification (i.e. underreporting). While the parent study included a qualitative component, it was not focused on sexual harassment, which may have helped contextualize findings. Finally, due to survey space constraints, we lack details on sexual harassment context (e.g., work, school, public, transit, home), and perpetrators (e.g., teacher, supervisor, stranger, family member). We note it is possible that the unwanted sexual attention occurred in the home, and our measure is not exclusive of partner harassment. We were also unable to assess how students were attending school (i.e., in person or virtually), which may have influenced the sexual harassment dynamics.

Despite these limitations, taken together, results suggest an underlying gendered risk context in diverse spaces,[7,5 35] which contributes to endemic GBV and permeates the lives of AGYW. Experiences of sexual harassment across public spaces (i.e., transit, school, and work) are a reflection of patriarchal social norms that govern where and when women can occupy space[8] and the consequences for entering a 'contested'[5] space. Other scholars have characterized environments of unwanted sexual attention and pressure as "coercive sexual environments" that are harmful in their own right, and simultaneously give rise to more egregious forms of GBV while reinforcing norms of tolerance, impunity and victim-blaming for such experiences.[6] 'Safety work',[5] or behaviors employed by women to avoid sexual harassment, such as limiting mobility,[11] prevent women and girls' full participation in society and can have long-term consequences for AGYW's educational attainment and economic opportunity and, in turn, their future health and well-being.[9 10] These consequences may be particularly acute for low-resourced AGYW or AGYW living in low-income environments.[35]

The momentum gained from increased attention to GBV in Kenya during the COVID-19 pandemic must be harnessed to concurrently increase focus on the burden of sexual harassment among AGYW in this

For peer review only - http://bmjopen.bmj.com/site/about/guidelines.xhtml

setting.[46] As the severity of the COVID-19 pandemic and associated restrictions continue to fluctuate, increased funding for the development and evaluation of interventions to prevent and address sexual harassment are needed. In an important first step, President Kenyatta has pledged significant funds to address GBV in Kenya[47] and the Government of Kenya has released a National Action plan for eliminating GBV in Kenya by 2026.[48] However, these efforts focus primarily on sexual harassment in the workplace[49] and should be expanded to include sexual harassment at school, on transit, and in other public spaces. Further, future interventions must focus on sexual harassment and its sequelae specifically among AGYW to mitigate the long-term impacts of sexual harassment on the social, economic, and physical well-being of this population. Finally, efforts must focus on the norms that perpetuate the gendered risk of violence and the potential for social norms change to reduce GBV perpetration and promote help-seeking.

For peer review only

Author contributions:
Study design and conceptualization: Decker, Gichangi, Thiongo
Protocol development (measures, safety protocol): Decker, Gichangi, Thiongo, Wamue-Ngare
Analysis: Williams, Bevilacqua
Writing: Bevilacqua, Williams
Editing and interpretation of data: Wamue-Ngare, Gichangi, Wood, Decker

Competing interests: None declared

Funding: This work was supported by the Bill & Melinda Gates Foundation [010481]. Under the Foundation grant conditions, a Creative Commons Attribution 4.0 Generic License has already been assigned to the Author Accepted Manuscript version that might arise from this submission.

Data Sharing Statement:
Data are available upon request from pmadata.org.

Research Ethics Approval:
Procedures were approved by the Ethics Review Committee at Kenyatta National Hospital/University of Nairobi (P310/06/2020) and the Institutional Review Boards at Johns Hopkins Bloomberg School of Public Health (IRB 00012952).

For peer review only

References

1. Kearl H. The facts behind the #MeToo movement: A national study on sexual harassment and assault: Stop Street Harassment, 2018.
2. Ranganathan M, Wamoyi J, Pearson I, et al. Measurement and prevalence of sexual harassment in low-and middle-income countries: a systematic review and meta-analysis. *BMJ Open* 2021;11(6):e047473. doi: 10.1136/bmjopen-2020-047473
3. Logan LS. Street harassment: Current and promising avenues for researchers and activists. *Sociology Compass* 2015;9(3):196-211. doi: https://doi.org/10.1111/soc4.12248
4. Fileborn B, O'Neill T. From "Ghettoization" to a field of its own: A comprehensive review of street harassment research. *Trauma, Violence, & Abuse* 2021:15248380211021608. doi: 10.1177/15248380211021608
5. Vera-Gray F, Kelly L. Contested gendered space: Public sexual harassment and women's work safety. In: Ceccato V, Nalla MK, eds. Crime and Fear in Public Places Towards Safe, Inclusive and Sustainable Cities. London: Routledge 2020:217-31.
6. Smith R, Gallagher M, Popkin S, et al. Coercive sexual environments: What MTO tells us about neighborhoods and sexual safety. *CityScape* 2014;16(1):85-112.
7. UN Women. Safe cities and safe public spaces: Global results report, 2017.
8. Gardner CB. Passing By: Gender and Public Harassment. Berkeley: University of California Press 1995:4.
9. Sifuna DN. A review of major obstacles to women's participation in higher education in Kenya. *Research in Post-Compulsory Education* 2006;11(1):85-105. doi: 10.1080/13596740500507995
10. Littleton H, Abrahams N, Bergman M, et al. Sexual assault, sexual abuse, and harassment: Understanding the mental health impact and providing care for survivors: An International Society for Traumatic Stress Studies Briefing Paper: International Society for Traumatic Stress Studies, 2018.
11. Gardner N, Cui J, Coiacetto E. Harassment on public transport and its impacts on women's travel behaviour. *Australian Planner* 2017;54(1):8-15. doi: 10.1080/07293682.2017.1299189
12. Eom E, Restaino S, Perkins AM, et al. Sexual harassment in middle and high school children and effects on physical and mental health. *Clinical Pediatrics* 2015;54(5):430-38. doi: 10.1177/0009922814553430
13. Houle JN, Staff J, Mortimer JT, et al. The impact of sexual harassment on depressive symtoms during the early occupational career. *Soc Ment Health* 2011;1(2):89-105. doi: 10.1177/2156869311416827
14. Chiodo D, Wolfe DA, Crooks C, et al. Impact of sexual harassment victimization by peers on subsequent adolescent victimization and adjustment: A longitudinal study. *Journal of Adolescent Health* 2009;45(3):246-52. doi: https://doi.org/10.1016/j.jadohealth.2009.01.006
15. Muasya J. Effects of sexual harassment on women students' access to opportunities and facilities: A case study of the University of Nairobi, Kenya. *Global Journal of Interdisciplinary Social Sciences* 2014;3(4):83-90.
16. United Nations. Prohibition of discrimination, harassment, including sexual harassment, and abuse of authority Secretary-General's Bulletin, 2008.
17. Abuya BA, Onsomu EO, Moore D. Educational challenges and diminishing family safety net faced by high-school girls in a slum residence, Nairobi, Kenya. *International Journal of*

For peer review only - http://bmjopen.bmj.com/site/about/guidelines.xhtml

Educational Development 2012;32(1):81-91. doi: https://doi.org/10.1016/j.ijedudev.2011.02.012
18. Loukaitou-Sideris A. Fear and safety in transit environments from the women's perspective. *Security Journal* 2014;27(2):242-56. doi: 10.1057/sj.2014.9
19. Mellgren C, Andersson M, Ivert A-K. "It Happens All the Time": Women's experiences and normalization of sexual harassment in public space. *Women & Criminal Justice* 2018;28(4):262-81. doi: 10.1080/08974454.2017.1372328
20. Bourgault S, Peterman A, O'Donnell M. Violence against women and children during COVID-19—one year on and 100 papers in. Violence Against Women and Children During COVID-19: Research Round Up. Washington, DC: Center for Global Development, 2021.
21. UN Women. Measuring the shadow pandemic: Violence against women during COVID-19, 2021.
22. World Health Organization. Health in 2015: from MDGs, millennium development goals to SDGs, sustainable development goals, 2015.
23. International Centre for Reproductive Health-Kenya (ICRHK) & PMA Agile. Nairobi Youth Respondent-Driven Sampling Survey: Final Report. 2020. Performance Monitoring for Action Technical Report. Baltimore, MD, USA: Bill & Melinda Gates Institute for Population and Reproductive Health, Johns Hopkins University Bloomberg School of Public Health, 2020.
24. Kenyatta U. Address to the Nation by h.e. Uhuru Kenyatta, c.g.h, President of the Republic of Kenya and Commander-in-Chief of the Defence Forces on COVID-19, commonly known as coronavirus at Harambee House, Nairobi on 15th March 2020 [Speech], 2020.
25. World Health Organization. Ethical and safety recommendations for intervention research on violence against women: building on lessons from the WHO publication putting women first: ethical and safety recommendations for research on domestic violence against women. Geneva, Switzerland: WHO, 2016
26. Center on Gender Equity and Health. Evidence-based Measures of Empowerment for Research on Gender Equality (EMERGE), 2017.
27. Operario D, Adler NE, Williams DR. Subjective social status: reliability and predictive utility for global health. *Psychology & Health* 2004;19(2):237-46. doi: 10.1080/08870440310001638098
28. Decker MR, Bevilacqua K, Wood SN, et al. Gender-based violence during COVID-19 among adolescent girls and young women in Nairobi, Kenya: a mixed-methods prospective study over 18 months. *BMJ global health* 2022;7(2):e007807.
29. Kenya National Bureau of Statistics, Kenya Ministry of Health, Kenya National AIDS Control Council, et al. Kenya Demographic and Health Survey 2014. Rockville, MD, USA, 2015.
30. Ministry of Labour and Social Protection of Kenya Department of Children's Services. Violence against Children in Kenya: Findings from a National Survey, 2019. Nairobi, Kenya, 2019.
31. Bhattacharjee P, Ma H, Musyoki H, et al. Prevalence and patterns of gender-based violence across adolescent girls and young women in Mombasa, Kenya. *BMC Women's Health* 2020;20(1):229. doi: 10.1186/s12905-020-01081-8

For peer review only

32. Kaltiala-Heino R, Fröjd S, Marttunen M. Sexual harassment victimization in adolescence: Associations with family background. *Child Abuse & Neglect* 2016;56:11-19. doi: https://doi.org/10.1016/j.chiabu.2016.04.005
33. Khoury-Kassabri M. Student victimization by educational staff in Israel. *Child Abuse & Neglect* 2006;30(6):691-707. doi: https://doi.org/10.1016/j.chiabu.2005.12.003
34. Khoury-Kassabri M, Benbenishty R, Avi Astor R, et al. The contributions of community, family, and school variables to student victimization. *American Journal of Community Psychology* 2004;34(3):187-204. doi: 10.1007/s10464-004-7414-4
35. Popkin SJ, Acs G, Smith R. Understanding how place matters for kids *Community Investments* 2010;22:23-26.
36. Koi VO, Auka J, Kilaha S. Perceived magnitude of sexual harassment in learning institutions: A case study of Kenya Medical Traning College, Nairobi. *International Academic Journal of Social Sciences and Education* 2018;2(1):65-73.
37. Kibunja BK, Musembi HM, Kimani RW, et al. Prevalence and effect of workplace violence against emergency nurses at a tertiary hospital in Kenya: A cross-sectional study. *Safety and Health at Work* 2021;12(2):249-54. doi: https://doi.org/10.1016/j.shaw.2021.01.005
38. Quaife M, van Zandvoort K, Gimma A, et al. The impact of COVID-19 control measures on social contacts and transmission in Kenyan informal settlements. *BMC Medicine* 2020;18(1):316. doi: 10.1186/s12916-020-01779-4
39. Piquero AR, Jennings WG, Jemison E, et al. Domestic violence during the COVID-19 pandemic - Evidence from a systematic review and meta-analysis. *Journal of Criminal Justice* 2021;74:101806. doi: https://doi.org/10.1016/j.jcrimjus.2021.101806
40. Pinchoff J, Austrian K, Rajshekhar N, et al. Gendered economic, social and health effects of the COVID-19 pandemic and mitigation policies in Kenya: evidence from a prospective cohort survey in Nairobi informal settlements. *BMJ Open* 2021;11(3):e042749. doi: 10.1136/bmjopen-2020-042749 [published Online First: 2021/03/05]
41. Roesch E, Amin A, Gupta J, et al. Violence against women during covid-19 pandemic restrictions. *BMJ* 2020;369:m1712. doi: 10.1136/bmj.m1712 [published Online First: 2020/05/10]
42. The Sexual Offenses Act. In: Kenya Po, ed. No 3 of 2006. Nairobi, Kenya: Government of Kenya, 2006.
43. The Sexual Offenses Act Revised Edition. In: Kenya AGo, ed. No 3 of 2006. Nairobi, Kenya: National Council for Law Reporting, 2009.
44. Dozens protest gender-based violence in Kenya. *La Prensa* 2022.
45. Rabin RF, Jennings JM, Campbell JC, et al. Intimate partner violence screening tools: a systematic review. *Am J Prev Med* 2009;36(5):439-45 e4. doi: 10.1016/j.amepre.2009.01.024 [published Online First: 2009/04/14]
46. Bhalla N. Kenya orders probe into rise in violence against women and girls during pandemic. *Reuters* 2020.
47. Bhalla N. Kenya president pledges millions to fight surge in gender violence. *Reuters* 2020.
48. UN Women Africa. Kenya's platform for action revealed ahead of Generation Equality Forum, 2021.
49. Republic of Kenya. Generation equality forum: Kenya's roadmap for advancing gender equality and ending all forms of gender based violence and female genital mutilation by 2026. Kenya: UN Women, International Center for Research on Women, United Nations Population Fund, 2021.

For peer review only - http://bmjopen.bmj.com/site/about/guidelines.xhtml

Figure 1. Trajectories of sexual harassment relative to and changes in frequency since the COVID-10 pandemic among adolescent girls and young women in Nairobi, Kenya in mid-2020 (n=612)

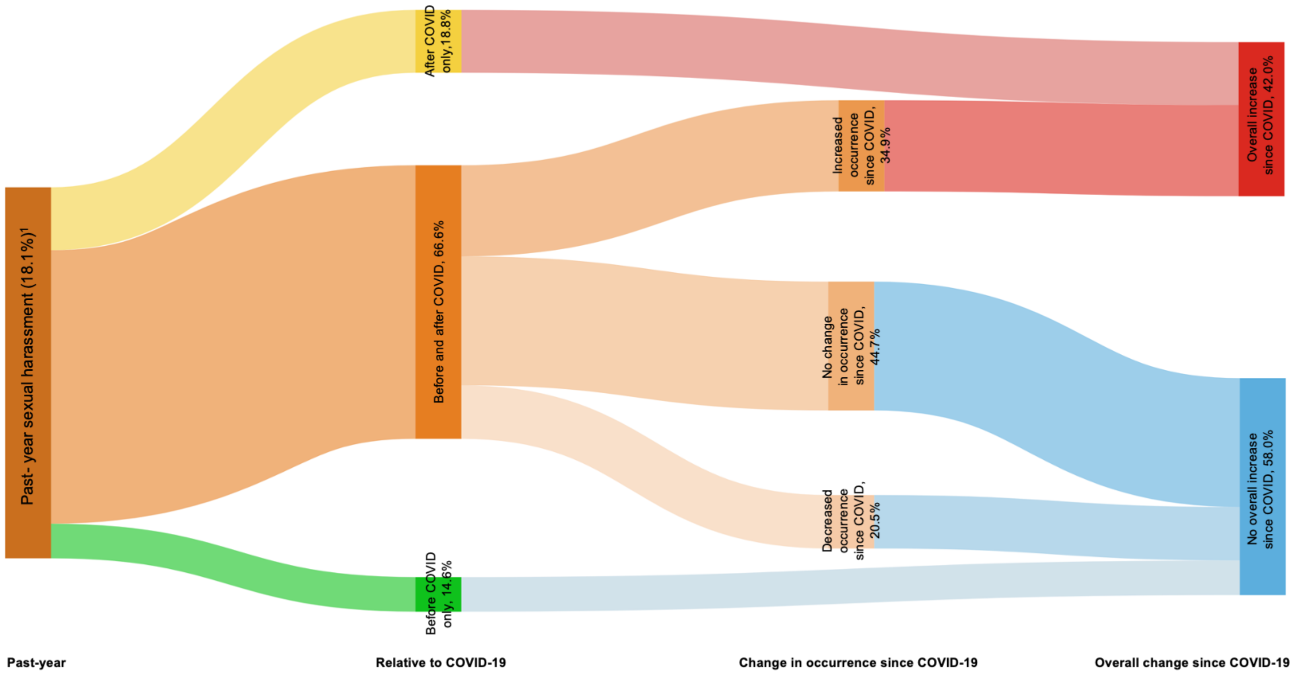

1. Among women reporting past-year sexual harassment in mid-2020

review only

STROBE Statement—Checklist of items that should be included in reports of ***cohort studies***

	Item No	Recommendation	Page No
**Title and abstract**	1	(*a*) Indicate the study's design with a commonly used term in the title or the abstract	1, 2
		(*b*) Provide in the abstract an informative and balanced summary of what was done and what was found	2
**Introduction**			
Background/rationale	2	Explain the scientific background and rationale for the investigation being reported	4-5
Objectives	3	State specific objectives, including any prespecified hypotheses	5
**Methods**			
Study design	4	Present key elements of study design early in the paper	5
Setting	5	Describe the setting, locations, and relevant dates, including periods of recruitment, exposure, follow-up, and data collection	5-6
Participants	6	(*a*) Give the eligibility criteria, and the sources and methods of selection of participants. Describe methods of follow-up	5
		(*b*) For matched studies, give matching criteria and number of exposed and unexposed	N/A
Variables	7	Clearly define all outcomes, exposures, predictors, potential confounders, and effect modifiers. Give diagnostic criteria, if applicable	6-7
Data sources/ measurement	8*	For each variable of interest, give sources of data and details of methods of assessment (measurement). Describe comparability of assessment methods if there is more than one group	6-7
Bias	9	Describe any efforts to address potential sources of bias	8
Study size	10	Explain how the study size was arrived at	7
Quantitative variables	11	Explain how quantitative variables were handled in the analyses. If applicable, describe which groupings were chosen and why	7
Statistical methods	12	(*a*) Describe all statistical methods, including those used to control for confounding	7-8
		(*b*) Describe any methods used to examine subgroups and interactions	N/A
		(*c*) Explain how missing data were addressed	N/A
		(*d*) If applicable, explain how loss to follow-up was addressed	7
		(*e*) Describe any sensitivity analyses	N/A
**Results**			
Participants	13*	(a) Report numbers of individuals at each stage of study—eg numbers potentially eligible, examined for eligibility, confirmed eligible, included in the study, completing follow-up, and analysed	7
		(b) Give reasons for non-participation at each stage	N/A
		(c) Consider use of a flow diagram	N/A
Descriptive data	14*	(a) Give characteristics of study participants (eg demographic, clinical, social) and information on exposures and potential confounders	8
		(b) Indicate number of participants with missing data for each variable of interest	N/A
		(c) Summarise follow-up time (eg, average and total amount)	N/A
Outcome data	15*	Report numbers of outcome events or summary measures over time	8

For peer review only - http://bmjopen.bmj.com/site/about/guidelines.xhtml

Main results	16	(*a*) Give unadjusted estimates and, if applicable, confounder-adjusted estimates and their precision (eg, 95% confidence interval). Make clear which confounders were adjusted for and why they were included	8-11
		(*b*) Report category boundaries when continuous variables were categorized	N/A
		(*c*) If relevant, consider translating estimates of relative risk into absolute risk for a meaningful time period	N/A
Other analyses	17	Report other analyses done—eg analyses of subgroups and interactions, and sensitivity analyses	N/A
**Discussion**			
Key results	18	Summarise key results with reference to study objectives	11-12
Limitations	19	Discuss limitations of the study, taking into account sources of potential bias or imprecision. Discuss both direction and magnitude of any potential bias	13
Interpretation	20	Give a cautious overall interpretation of results considering objectives, limitations, multiplicity of analyses, results from similar studies, and other relevant evidence	11-12
Generalisability	21	Discuss the generalisability (external validity) of the study results	13
**Other information**			
Funding	22	Give the source of funding and the role of the funders for the present study and, if applicable, for the original study on which the present article is based	2 (abstract)

*Give information separately for exposed and unexposed groups.

Note: An Explanation and Elaboration article discusses each checklist item and gives methodological background and published examples of transparent reporting. The STROBE checklist is best used in conjunction with this article (freely available on the Web sites of PLoS Medicine at http://www.plosmedicine.org/, Annals of Internal Medicine at http://www.annals.org/, and Epidemiology at http://www.epidem.com/). Information on the STROBE Initiative is available at http://www.strobe-statement.org.

For peer review only

For peer review only - http://bmjopen.bmj.com/site/about/guidelines.xhtml

BMJ Open

Sexual harassment before and during the COVID-19 pandemic among adolescent girls and young women (AGYW) in Nairobi, Kenya: a cross-sectional study

Journal:	*BMJ Open*
Manuscript ID	bmjopen-2022-066777.R1
Article Type:	Original research
Date Submitted by the Author:	19-Sep-2022
Complete List of Authors:	Bevilacqua, Kristin; Johns Hopkins University Bloomberg School of Public Health, Department of Population, Family and Reproductive Health<br>Williams, A; Johns Hopkins University Bloomberg School of Public Health, Department of Population, Family and Reproductive Health<br>Wood, Shannon; Johns Hopkins University Bloomberg School of Public Health, Department of Population, Family and Reproductive Health; Bill & Melinda Gates Institute for Population and Reproductive Health, Department of Population, Family and Reproductive Health, Johns Hopkins Bloomberg School of Public Health<br>Wamue-Ngare, G; Kenyatta University, Department of Sociology, Gender and Development Studies; Kenyatta University, Women’s Economic Empowerment Hub<br>Thiongo, Mary; International Centre for Reproductive Health Kenya<br>Gichangi, P; International Centre for Reproductive Health Kenya; Ghent University, Department of Public Health and Primary Care<br>Decker, Michele; Johns Hopkins University Bloomberg School of Public Health, Department of Population, Family and Reproductive Health; Bill & Melinda Gates Institute for Population and Reproductive Health, Department of Population, Family and Reproductive Health, Johns Hopkins Bloomberg School of Public Health,
**Primary Subject Heading**:	Global health
Secondary Subject Heading:	Epidemiology, Public health, Sexual health
Keywords:	COVID-19, PUBLIC HEALTH, EPIDEMIOLOGY

SCHOLARONE™
Manuscripts

I, the Submitting Author has the right to grant and does grant on behalf of all authors of the Work (as defined in the below author licence), an exclusive licence and/or a non-exclusive licence for contributions from authors who are: i) UK Crown employees; ii) where BMJ has agreed a CC-BY licence shall apply, and/or iii) in accordance with the terms applicable for US Federal Government officers or employees acting as part of their official duties; on a worldwide, perpetual, irrevocable, royalty-free basis to BMJ Publishing Group Ltd ("BMJ") its licensees and where the relevant Journal is co-owned by BMJ to the co-owners of the Journal, to publish the Work in this journal and any other BMJ products and to exploit all rights, as set out in our licence.

The Submitting Author accepts and understands that any supply made under these terms is made by BMJ to the Submitting Author unless you are acting as an employee on behalf of your employer or a postgraduate student of an affiliated institution which is paying any applicable article publishing charge ("APC") for Open Access articles. Where the Submitting Author wishes to make the Work available on an Open Access basis (and intends to pay the relevant APC), the terms of reuse of such Open Access shall be governed by a Creative Commons licence – details of these licences and which Creative Commons licence will apply to this Work are set out in our licence referred to above.

Other than as permitted in any relevant BMJ Author's Self Archiving Policies, I confirm this Work has not been accepted for publication elsewhere, is not being considered for publication elsewhere and does not duplicate material already published. I confirm all authors consent to publication of this Work and authorise the granting of this licence.

For peer review only

Sexual harassment before and during the COVID-19 pandemic among adolescent girls and young women (AGYW) in Nairobi, Kenya: a cross-sectional study

Bevilacqua KG[1] kbevila1@jhu.edu; 1-516-297-4305
Williams A[1] awill137@jhu.edu; 1-207-245-0551
Wood SN[1,2] swood@jhu.edu; 1-410-955-0000
Wamue-Ngare G[3,4] wamue.grace@ku.ac.ke; 8710901-19
Thiongo M[5] maryt@icrhk.org; 254 722 208 652
Gichangi P[5,6,7] gichangip2015@gmail.com; 254 722 208 652
Decker MR[1,2*] mdecker@jhu.edu; 1-410-502-2747

[1] Department of Population, Family and Reproductive Health, Johns Hopkins University Bloomberg School of Public Health, Baltimore, Maryland, USA.
[2] Bill & Melinda Gates Institute for Population and Reproductive Health, Department of Population, Family and Reproductive Health, Johns Hopkins University Bloomberg School of Public Health, Baltimore, Maryland USA.
[3] Department of Sociology, Gender and Development Studies, Kenyatta University, Nairobi, Kenya.
[4] Women's Economic Empowerment Hub, Kenyatta University, Nairobi, Kenya.
[5] International Centre for Reproductive Health-Kenya, Nairobi, Kenya.
[6] Technical University of Mombasa, Mombasa, Kenya.
[7] Department of Public Health and Primary Care, Faculty of Medicine and Health Sciences, Ghent University, Belgium

*Corresponding author:
Michele Decker
615 N Wolfe St
Baltimore, MD 21205
mdecker@jhu.edu

Word count: 3,292
Tables: 3
Figures: 1
References: 49
Abstract word count: 299/300

For peer review only

For peer review only - http://bmjopen.bmj.com/site/about/guidelines.xhtml

Abstract

Objectives: Sexual harassment among adolescent girls and young women (AGYW) is a prevalent and understudied form of gender-based violence (GBV) with negative impacts on health and well-being. The COVID-19 pandemic raised global concern about GBV within homes; less is known about how it affected GBV in public spaces.

Methods: Present analyses utilize cross-sectional data from a cohort of adolescents and young adults residing in Nairobi, Kenya, restricted to female participants. Data was collected August-October 2020 via phone after implementation of COVID-19 restrictions. Prevalence of past-year sexual harassment and harassment relative to COVID-19 restrictions were calculated for overall sample, and by individual, household, and pandemic-related factors. Multivariate negative binomial regression models examine correlates of 1) past-year sexual harassment and 2) increases in sexual harassment relative to COVID-19 restrictions.

Results: Overall, 18.1% of AGYW experienced past-year sexual harassment at the 2020 survey. Among this group, 14.6% experienced sexual harassment pre-COVID-19 only, 18.8% after only, and 66.6% at both timepoints. Among the latter group, 34.9% reported more occurrences following COVID-19 restrictions, 20.6% reported less occurrences, and 44.7% reported no change in occurrence. Overall, 42.0% of AGYW experienced an increase in sexual harassment while 58.0% experienced no increase since COVID-19. In adjusted models, past-year sexual harassment was associated with higher educational attainment (aRR: 2.11; 95% CI: 1.27, 3.52) and inability to meet basic financial needs (aRR: 1.67; 95% CI: 1.05, 2.66). Increased sexual harassment since COVID-19 was associated with having full control to leave the home (aRR: 1.69; 95% CI: 1.00, 2.90).

Conclusions: Sexual harassment among AGYW in Nairobi, Kenya was prevalent before and during COVID-19 restrictions. Safety in public spaces remains a highly gendered issue that impacts women's safety and ability to participate in public life. Prevention and support services to address sexual harassment remain an important element in ensuring safe, sustainable public spaces.

Strengths and limitations:

- This is the first study to examine prevalence of sexual harassment in relation to the COVID-19 pandemic among adolescent girls and young women (AGYW) in Nairobi, Kenya.
- We focus on AGYW, a key developmental time-period during which experiences of violence may have long-term consequences for health, well-being, and future risk of GBV.
- Cross-sectional, retrospective data limits our ability to explore causal relationships.
- Use of a single-item outcome measure may have reduced sensitivity, increasing risk of misclassification (i.e., underreporting) of sexual harassment.

For peer review only

BACKGROUND

Global awareness of sexual harassment has risen steadily since 2017, stemming from the global #MeToo, Times Up, and Generation Equality movements. Yet relative to other leading forms of gender-based violence (GBV), sexual harassment remains understudied. Sexual harassment can include staring, cat calling, forced conversation, and/or stalking by either strangers, peers, acquaintances, or authority figures, and often occurs in public spaces, such as on the street or in workplace or educational settings but can also occur in the home and other private spaces.[1] A recent systematic review meta-analysis found wide-ranging prevalence estimates from 0.6%–26.1%, globally,[2] potentially reflecting differences in the conceptualization and measurement of sexual harassment, which occurs across diverse spaces and perpetrators.[3-5] Sexual harassment can serve as a potent reminder of underlying gender imbalances and predatory sexual climates known as coercive sexual environments,[6] which disproportionately burden adolescent girls and young women (AGYM).[5 7 8]

Experiences of sexual harassment during adolescence and young adulthood can have serious consequences for the health and well-being of AGYW. Sexual harassment contributes to increased risk of work and school absenteeism, compromising AGYW's future economic stability.[9 10] For example, harassment experienced on public transportation may compromise AGYW's labor or educational participation and advancement if they are unable to travel safely.[7 11] Experiences of sexual harassment are also associated with a host of negative mental health outcomes, including stress, anxiety, and fear.[10 12 13] Further, sexual harassment among AGYW is associated with elevated risk of future harassment, as well as physical and sexual violence victimization,[14] reflecting embedded social/environmental risks. Despite its consequences, there is a dearth of research aimed at understanding risk factors for sexual harassment among AGYW,[4] and existing research is largely concentrated in high-income countries or among high-income subgroups within low- and middle-income countries (LMICs).[2]

Mirroring gaps in sexual harassment research in LMICs, few studies have explored sexual harassment specifically within Kenya.[15 16] The limited available literature is qualitative and offers useful insight into sexual harassment among AGYW. In one case study at University of Nairobi, women described sexual harassment as a main barrier to feeling comfortable taking certain classes, using university toilets, and accessing certain spaces on campus, such as the library.[15] Another study among high school AGYW in Nairobi's urban settlements highlighted how sexual harassment experienced in transit to and from school often escalated from verbal harassment to unwanted touching, and when men's attention was not reciprocated, it could lead to violence.[17] Though quantitative assessments of sexual harassment among

For peer review only - http://bmjopen.bmj.com/site/about/guidelines.xhtml

AGYW in Kenya are not currently available, qualitative findings align with experiences of sexual harassment among this population in other settings and highlight the gendered risks AGYW face across public contexts such as school, work, and public transportation.[18,19]

The public nature of sexual harassment distinguishes it from other forms of GBV like intimate partner violence, which occur more often in private spaces. The COVID-19 pandemic raised global concern for GBV in *private* spaces due to increased time in the home with potentially abusive partners,[20] however, the potential impacts of changes to AGYW's mobility and time spent *outside of the home* on experiences of GBV are less clear. For example, closures and mobility restrictions due to pandemic policies may decrease sexual harassment risk in some settings like schools but could increase risk of sexual harassment among AGYW traveling in spaces made more isolated by pandemic restrictions. Early evidence of these dynamics has been observed in Kenya; a 2021 UN report on violence against women during the pandemic showed that 55% of women in Kenya reported that COVID-19 made them feel less safe walking alone at night and 81% perceived an increase in sexual harassment since the start of the pandemic.[21] However, to our knowledge, no studies have explored the prevalence of sexual harassment or risk factors associated with potential COVID-related increases among the priority population of AGYW in Kenya.

The United Nations' Sustainable Development Goal (SDG) 11 calls for the promotion of safe, resilient, and sustainable cities and settlements.[22] Efforts to achieve this goal must employ a gendered perspective to better understand and address the violence and hostility experienced by girls and women in public spaces.[7] To this end, the present study utilizes cross-sectional data from a cohort of youth in Nairobi, Kenya to examine (1) the prevalence of past-year sexual harassment among a sample of AGYW overall, and relative to the implementation of COVID-19 restrictions, (2) factors associated with any past-year sexual harassment, and (3) factors associated with increased sexual harassment relative to COVID-19 restrictions.

METHODS

Study population

The study population is a subsample drawn from the Nairobi Youth Respondent Driven Sampling Survey (YRDSS), an ongoing cohort study of adolescents and young adults first recruited pre-pandemic, in 2019. Eligible participants were age 15-24 years, unmarried, and residing in Nairobi for at least one year at the time of enrollment. In August 2019, 1,357 young men and young women were surveyed, 95% of whom (1,293/1,357) consented for recontact. At 12-month follow-up (August-October 2020) 1,217 (94%) were successfully recontacted and consented and completed the follow-up survey. Additional sampling and recruitment details can be found elsewhere.[23] Sexual harassment experiences and their timing relative to

For peer review only

COVID-19 pandemic restrictions were assessed at the 2020 survey wave only; the present analytic sample is restricted to all female participants in the 2020 survey (n=612).

Data collection

COVID-19 restrictions were first implemented in Kenya in March 2020 when President Kenyatta ordered the closing of schools and non-essential workplaces and barred travel from countries with reported cases of COVID-19, among other restrictions.[24] Due to the implementation of COVID-19 restrictions, trained resident enumerators (REs) conducted data collection by phone in either English or Swahili. Oral consent was recorded electronically via OpenDataKit The Ethics Review Committee (ERC) at Kenyatta National Hospital/University of Nairobi. The Institutional Review Board at Johns Hopkins Bloomberg School of Public Health waived parental consent for this study and approved use of oral consent processes. All consent procedures aligned with ethical best practices for sensitive topics, including specialized training, privacy protections (auditory privacy screener and protocol), and provision of resource referrals. Participants who consented for recontact in 2019 were consented prior to the 2020 follow-up. All data were collected in accordance with best practices for GBV research and RE's received specialized training specific to GBV protections.[25] To ensure privacy and safety for GBV and other sensitive topics during remote data collection, RE's confirmed participant safety and privacy and rescheduled as needed. GBV support services were provided to all participants within a larger list of supports to minimize risk. Participants received 500 KES or $5 USD per survey completed.

Research Ethics Approval

Procedures were approved by the Ethics Review Committee at Kenyatta National Hospital/University of Nairobi (P310/06/2020) and the Institutional Review Boards at Johns Hopkins Bloomberg School of Public Health (IRB 00012952).

Measures

Primary outcomes

Past-year sexual harassment was assessed with a single item adapted from seminal research on coercive sexual environments:[6] "In the past 12-months, have you experienced unwanted sexual attention or harassment such as verbal comments, staring or leering, or unwanted physical contact like groping or grabbing?" Response options included: never, once, a few times, often, or no response. Among AGYW who reported any past-year sexual harassment, participants were asked to report timing relative to the COVID-19 pandemic restrictions (March 2020); "Have experiences of unwanted sexual attention or harassment happened before COVID-19 restrictions only, since COVID-19 restrictions only, or both?"

For peer review only - http://bmjopen.bmj.com/site/about/guidelines.xhtml

AGYW who reported sexual harassment both before and after COVID-19 restrictions described changes in occurrences; "Have experiences of sexual harassment changed since the COVID-19 restrictions began?" (response options: increased a lot, increased somewhat, no change, decreased somewhat, decreased a lot). To maximize power for analyses, increased sexual harassment since the pandemic, among those who experienced past year sexual harassment, was modeled dichotomously: AGYW were classified as having increased sexual harassment if they first started experiencing sexual harassment after pandemic restrictions began or if they experienced sexual harassment both before and after pandemic restrictions but reported an increase in occurrence since the start of the pandemic. AGYW were classified as no increase in sexual harassment if they experienced sexual harassment only before the pandemic or if they experienced sexual harassment before and after pandemic restrictions but reported a decrease in occurrence during the pandemic or if they experienced sexual harassment before and after pandemic restrictions but reported no change in occurrence relative to the pandemic.

Independent variables

Individual, household, and pandemic-related covariates were measured in 2020. At the individual level, demographic assessments including age, educational attainment, current school enrollment status, and marital status were obtained. AGYW's control over the decision to leave the house and over their own earnings was assessed as part of a broader measure on personal agency via 4-point Likert scale, with categories collapsed for analysis (0="less than full control," or 1="full control").[26] Household-level covariates were comprised of subjective household socioeconomic status (SES)[27] and household composition (response options: 0= "living alone," 1= "living with parents with or without others," 2= "living with partner with or without others," 3= "living with others"). The pandemic-related covariate focused on inability to meet basic needs since the start of the COVID-19 pandemic, assessed via 4-point Likert scale and dichotomized for analysis (0= "basic financial needs met," or 1= "basic financial needs not met").

Analysis

We first calculated the proportion of AGYW who experienced sexual harassment in the 12 months preceding the survey and the timing of these experiences relative to the COVID-19 restrictions (only before, only after, or both). Among those who reported sexual harassment both before and after pandemic restriction initiation, we characterized changes in occurrences relative to COVID-19 restrictions (i.e., no change, decreased occurrence, or increased occurrence) and overall changes in sexual harassment since COVID-19 restrictions (i.e., increase or no increase). We examined each past-year sexual harassment and overall increased sexual harassment since COVID-19 restrictions by demographics, with differences

For peer review only - http://bmjopen.bmj.com/site/about/guidelines.xhtml

assessed via design-based F-statistics. Sexual harassment relative to COVID-19 pandemic restrictions among AGYW who experienced past-year sexual harassment (n=118) were visualized using a Sankey diagram (Figure 1).

Based on unadjusted bivariate associations, multivariate negative binomial regression models were constructed to examine correlates of 1) past-year sexual harassment and 2) increases in sexual harassment relative to COVID-19 pandemic restrictions, respectively. All analyses were conducted using Stata 15.1 (College Station, TX) with statistical significance set a priori at $p<0.05$; threshold for non-significant trend set at $p<0.10$. Sampling weights accommodate the RDS study design using RDS-II (Volz-Heckathorn) weights, and modest adjustments for post-estimation and loss-to-follow-up. Statistical testing accounts for survey weights and complex survey design.

Patient and Public Involvement

This community-engaged study incorporated community input throughout its inception, implementation, and dissemination. During its inception, key stakeholders from community-based, youth-serving organizations consulted on study recruitment, survey design, and data collection procedures. Trained RE's were recruited from study communities and provided integral input on survey design and data collection. Findings have been disseminated among key stakeholders, including government representatives, community and faith leaders, community-based organizations, and youth leaders.

RESULTS

Overall, 18.1% of AGYW reported past-year sexual harassment (Table 1). Among those who experienced past-year sexual harassment, 66.6% experienced harassment both before and after COVID-19 restrictions (March 2020), 14.6% experienced harassment only before and 18.8% experienced harassment only after pandemic restrictions. Among those endorsing sexual harassment both before and after COVID-19 restrictions, 44.7% experienced no change in occurrence, 34.9% experienced an increase in occurrence and 20.5% experienced a decrease in occurrence. Among AGWY who experienced any past-year sexual harassment, 42.0% experienced an overall increase relative to COVID-19 (i.e., new or increased sexual harassment since March 2020) (Figure 1).

Table 1: Sexual harassment prevalence, timing and changes relative to COVID-19 pandemic restrictions among AGYW, 2020 (n=612)

	weighted % (n)
Experienced in past 12 months	18.1 (118)
Timing relative to COVID-19 restrictions; among those who	

For peer review only - http://bmjopen.bmj.com/site/about/guidelines.xhtml

experienced past-year sexual harassment (n=118)	
Pre-COVID-19 only	14.6 (11)
Post- COVID-19 only	18.8 (20)
Both time periods	66.6 (87)
Changes in occurrence since COVID-19 restrictions; among those who experienced sexual harassment both prior to and during COVID-19 restrictions (n=87)	
No change	44.7 (41)
Decreased occurrence	20.5 (17)
Increased occurrence	34.9 (29)
Overall increase in sexual harassment since COVID-19 restrictions began, among those who experienced past-year sexual harassment+ (n=118)	
Increase	42.0 (49)
No increase	58.0 (69)

+new or increased sexual harassment since March 2020

peer review only

Correlates of Past-year Sexual Harassment

In bivariate analyses, past-year sexual harassment was more prevalent among AGYW with greater educational attainment (college/university vs. secondary or lower; 38.5% vs. 15.9%; p<0.01) (Table 2), whereas not being enrolled in school at the time of the survey was marginally associated with past-year sexual harassment (20.9% vs. 14.2%; p=0.11). AGYW who were unable to meet their basic financial needs since COVID-19 were more likely to experience past-year sexual harassment than those able to meet their basic financial needs (22.6% vs. 12.9%; p=0.02). In adjusted multivariate negative binomial models, inability to meet basic financial needs remained significantly associated with past-year sexual harassment (aRR: 1.67, 95% CI: 1.05, 2.66), as did higher educational attainment (aRR: 2.11, 95% CI: 1.27, 3.52) (Table 2).

Table 2: Factors associated with past-year sexual harassment among AGYW, weighted

	Sample (n=612)	Past-year sexual harassment		
	col%	row%	p-value+	aRR (95% CI)++
**Overall**	--	18.1	--	--
**Age group**			0.68	
16-20 years	42.6	19.1		--
20-26 years	57.4	17.3		--
**Highest level of education completed**			<0.01**	
Secondary or lower	90.4	15.9		ref
College / University	9.6	38.5		2.11** (1.27, 3.52)
**Currently in school**			0.11	
No	58.2	20.9		ref
Yes	41.8	14.2		0.82 (0.48, 1.40)
**Marital status**			0.46	

Unmarried	90.2	17.6		--
Married	9.8	22.6		--
**Personal control over decision to leave house**			0.85	
Less than full control	60.0	18.5		--
Full control	40.0	17.6		--
**Control over own earnings**			0.29	
Less than full control	71.7	16.7		--
Full control	28.3	21.7		--
**Relative household SES tertile**			0.41	
Lowest	37.0	14.4		--
Middle	22.2	19.3		--
Highest	40.7	20.8		--
**Household composition**			0.17	
Live alone	6.3	20.0		--
Lives with parent(s), with or without other(s)	66.4	17.2		--
Lives with partner with or without other(s), excluding parent(s)	10.0	30.7		--
Live with others	17.2	13.4		--
**Ability to meet basic financial needs since Covid-19**			0.02*	
Basic financial needs met	46.6	12.9		ref
Basic financial needs unmet	53.4	22.6		1.67* (1.05, 2.66)

+P-value for the design-based F statistic bivariate testing
++aRR (adjusted risk ratio) generated through multivariate negative binomial regression with past-year sexual harassment as the dependent variable, among full sample (n=612); all variables listed included in specification accounting for robust standard error clustering by node; weighted
*** p<0.001, ** p<0.01, * p<0.05

Correlates of Overall Increase in Sexual Harassment Since COVID-19

Among AGYW who experienced any past-year sexual harassment, in bivariate analyses, those currently enrolled in school were more likely to experience increased sexual harassment since the start of the COVID-19 restrictions compared to those not currently enrolled in school (59.0% vs. 33.7%; p=0.05). Having full control over one's decision to leave the home was marginally bivariately associated with increased sexual harassment (57.4% vs. 32.2%; p=0.06) (Table 3). In adjusted multivariate negative binomial models, full control to leave the home was associated with overall increased sexual harassment since the start of COVID-19 restrictions (aRR: 1.69; 95% CI: 1.00,2.90) (Table 3).

Table 3: Factors associated with increased sexual harassment since COVID-19 restrictions, among AGYW with past-year sexual harassment, weighted

	Sample (n=118)	Increased sexual harassment since Covid		
	col%	row%	p-value+	aRR (95% CI)++
**Overall**	--	42.0	--	--
**Age group**			0.38	
16-20 years	45.0	48.3		--
20-26 years	55.0	36.8		--
**Highest level of education completed**			0.60	
Secondary or lower	79.5	40.4		--

For peer review only

For peer review only - http://bmjopen.bmj.com/site/about/guidelines.xhtml

College / University	20.5	48.1		--
**Currently in school**			0.05*	
No	67.3	33.7		ref
Yes	32.7	59.0		1.66 (0.98, 2.80)
**Marital status**			0.66	
Unmarried	87.8	43.0		--
Married	12.2	35.1		--
**Personal control over decision to leave house**			0.06	
Less than full control	61.2	32.2		ref
Full control	38.8	57.4		1.69* (1.00, 2.90)
**Control over own earnings**			0.26	
Less than full control	66.2	46.5		--
Full control	33.8	33.2		--
**Relative household SES tertile**			0.97	
Lowest	29.6	43.3		--
Middle	23.6	43.3		--
Highest	46.8	40.5		--
**Household composition**			0.17	
Live alone	7.0	16.6		--
Lives with parent(s), with or without other(s)	63.3	44.4		--
Lives with partner with or without other(s), excluding parent(s)	17.0	25.9		--
Live with others	12.8	65.1		--
**Ability to meet basic financial needs since Covid-19**			0.59	
Basic financial needs met	33.3	37.2		--
Basic financial needs unmet	66.7	44.4		--

$^{+}$P-value for the design-based F statistic bivariate testing
$^{++}$aRR (adjusted risk ratio) generated through multivariate negative binomial regression with increased sexual harassment since COVID-19 restrictions as the dependent variable, among those who experienced past-year sexual harassment (n=118); all variables listed included in specification accounting for robust standard error clustering by node; weighted
*** p<0.001, ** p<0.01, * p<0.05

DISCUSSION

Sexual harassment was pervasive among AGYW in our sample, with 18.1% reporting experiences of sexual harassment in the past year. Among AGYW experiencing past-year sexual harassment, the majority reported those experiences occurring both before and after the start of COVID-19 pandemic restrictions. Within the subset of AGYW who experienced sexual harassment before and after COVID-19 restrictions were implemented, most (44.7%) experienced no change in occurrence of sexual harassment after the start of the pandemic. Overall, 42.0% experienced an increase in sexual harassment since the start of COVID-19 pandemic restrictions. The high prevalence of sexual harassment prior to the start of the pandemic and sustained or increased occurrence of such violence echoes the burdens of other forms of GBV in Kenya[28-31] and underscores the need for attention to sexual harassment and its impacts on health and well-being among AGYW in this setting.

For peer review only

Past-year sexual harassment was associated with AGYW's inability to meet their basic financial needs. These results support a growing body of research that has found lower socioeconomic status to be associated with increased risk of sexual harassment,[32] including during adolescence.[33-36] Interestingly, past-year sexual harassment was also associated with greater educational attainment, perhaps due to sexual harassment experienced in the school/university[37-40] or workplace settings.[41 42] We note that AGYW who were *not currently* enrolled in school during the COVID-19 pandemic were marginally significantly more likely to report past-year sexual harassment. These results suggest that young women who left school, potentially due to the pandemic, and more educated women who have already entered the workforce may have heightened risk. Results reflect the ways in which both *lower* socioeconomic status and *higher* educational attainment may contribute to increased time in public spaces, either out of financial necessity or workplace opportunities afforded by an advanced degree and suggest that greater access to educational and economic resources may not be protective against sexual harassment. Further, the increased risk associated with school enrollment suggests that initiatives to keep AGYW in school may not protect them from sexual harassment and that further intervention to reduce perpetration in school settings is urgently needed. Adolescence and young adulthood mark pivotal developmental junctures during which educational and economic opportunities shape future health and well-being. While public spaces remain unsafe for AGYW, their ability to participate in education or work will continue to be compromised, compounding their socioeconomic vulnerability and risk of future violence.

A growing body of literature has documented the ways in which pandemic-related restrictions and psychosocial stressors have contributed to increased GBV risk globally.[20 28 32] In contrast to more private forms of GBV like IPV, sexual harassment is often perpetrated in public spaces, making the potential impact of COVID-related restrictions and stressors less clear. The majority of AGYW who had ever experienced sexual harassment did so both before and after the start of the pandemic, suggesting that for most AGYW, pandemic-related restrictions had minimal impact on their risk of sexual harassment, perhaps reflecting economic realities that prevented many Kenyans from remaining home despite stay-at-home orders.[43] In adjusted models, personal control to leave the home was significantly associated with overall increased sexual harassment since the start of the pandemic among AGYW, and current school enrollment was marginally associated with increased sexual harassment. These results underscore the ways in which mobility restrictions may have presented a double edged sword for AGYW, whereby remaining at home may have placed them at greater risk of IPV or violence perpetrated by family members,[28 44-46] and leaving the home may have made them more vulnerable to sexual harassment in public spaces like work and transit.

For peer review only

Unfortunately, the current policy landscape does not reflect the realities of AGYW at risk for sexual harassment. The Sexual Offenses Act, passed by the Government of Kenya in 2006, defines sexual harassment as persistent sexual advances or requests made by *any person in a position of authority or holding public office*.[47] The law's narrow definition fails to address harassment perpetrated by peers or coworkers, in informal work settings, or in public. Notably, penal code 144(3), the part of the Act which prohibited insult to women's modesty or utterances of words or sounds and gestures that, "intrude upon the privacy of the woman or girl,"[47] but fell short of labeling such behavior as sexual harassment, has since been repealed.[48] While current policies fall short, public advocacy efforts and attention surrounding sexual harassment are mounting. For example, when a young woman was harassed and assaulted by boda boda (motorcycle) drivers in March 2022, large public protests erupted.[49] Increased research is urgently needed to inform policy and intervention to address the unique aspects of sexual harassment experienced during adolescence and young adulthood, including age-specific risk factors for perpetration and victimization as well as health impacts.

Results must be understood in the context of the study's limitations. Cross-sectional, retrospective data was obtained at a single point in time and limits our ability to explore causal relationships. Surveys were conducted via mobile phone, which may have resulted in a study sample of more well-resourced youth; however, post-estimation weights improve generalizability to the overall youth population in Nairobi. To minimize participant fatigue, a single item assessed sexual harassment, which may be less sensitive,[50] increasing risk of misclassification (i.e. underreporting). For example, one study that measured more specific forms of sexual harassment in a similar settings found higher prevalence of such violence both before and after the COVID-19 pandemic.[32] While the parent study included a qualitative component, it was not focused on sexual harassment, which may have helped contextualize findings. Finally, due to survey space constraints, we lack details on sexual harassment context (e.g., work, school, public, transit, home), perpetrators (e.g., teacher, supervisor, stranger, family member), and severity (e.g., level of threat, aggression, and harm incurred). We note it is possible that the unwanted sexual attention occurred in the home, and our measure is not exclusive of partner harassment. We were also unable to assess how students were attending school (i.e., in person or virtually), which may have influenced the sexual harassment dynamics.

Despite these limitations, taken together, results suggest an underlying gendered risk context in diverse spaces,[7,5 36] which contributes to endemic GBV and permeates the lives of AGYW. Experiences of sexual harassment across public spaces (i.e., transit, school, and work) are a reflection of patriarchal social norms that govern where and when women can occupy space[8] and the consequences for entering a 'contested'[5]

For peer review only - http://bmjopen.bmj.com/site/about/guidelines.xhtml

space. Other scholars have characterized environments of unwanted sexual attention and pressure as "coercive sexual environments" that are harmful in their own right, and simultaneously give rise to more egregious forms of GBV while reinforcing norms of tolerance, impunity and victim-blaming for such experiences.[6] 'Safety work,'[5] or behaviors employed by women to avoid sexual harassment, such as limiting mobility,[11] prevent women and girls' full participation in society and can have long-term consequences for AGYW's educational attainment and economic opportunity and, in turn, their future health and well-being.[9] [10] These consequences may be particularly acute for low-resourced AGYW or AGYW living in low-income environments.[36]

The momentum gained from increased attention to GBV in Kenya during the COVID-19 pandemic must be harnessed to concurrently increase focus on the burden of sexual harassment among AGYW in this setting.[51] As the severity of the COVID-19 pandemic and associated restrictions continue to fluctuate, increased funding for the development and evaluation of interventions to prevent and address sexual harassment are needed. In an important first step, President Kenyatta has pledged significant funds to address GBV in Kenya[52] and the Government of Kenya has released a National Action plan for eliminating GBV in Kenya by 2026.[53] However, these efforts focus primarily on sexual harassment in the workplace[54] and should be expanded to include sexual harassment at school, on transit, and in other public spaces. Further, future interventions must focus on sexual harassment and its sequelae specifically among AGYW to mitigate the long-term impacts of sexual harassment on the social, economic, and physical well-being of this population. Finally, efforts must focus on the norms that perpetuate the gendered risk of violence and the potential for social norms change to reduce GBV perpetration and promote help-seeking.

For peer review only

Author contributions:
MRD, PG, and MT conceptualized and designed the present study. Protocol development, including measures and safety protocol, was completed by MRD, PG, MT, and GWN. Analysis was conducted by AW and KGB. Manuscript preparation and writing were completed by KGB and AW. GWN, PG, SNW and MRD edited and interpreted the data.

Competing interests: None declared

Funding: This work was supported by the Bill & Melinda Gates Foundation [010481]. Under the Foundation grant conditions, a Creative Commons Attribution 4.0 Generic License has already been assigned to the Author Accepted Manuscript version that might arise from this submission.

Data Sharing Statement:
Data are available upon request from pmadata.org.

Research Ethics Approval:
Procedures were approved by the Ethics Review Committee at Kenyatta National Hospital/University of Nairobi (P310/06/2020) and the Institutional Review Boards at Johns Hopkins Bloomberg School of Public Health (IRB 00012952).

References

For peer review only

1. Kearl H. The facts behind the #MeToo movement: A national study on sexual harassment and assault: Stop Street Harassment, 2018.
2. Ranganathan M, Wamoyi J, Pearson I, et al. Measurement and prevalence of sexual harassment in low-and middle-income countries: a systematic review and meta-analysis. *BMJ Open* 2021;11(6):e047473. doi: 10.1136/bmjopen-2020-047473
3. Logan LS. Street harassment: Current and promising avenues for researchers and activists. *Sociology Compass* 2015;9(3):196-211. doi: https://doi.org/10.1111/soc4.12248
4. Fileborn B, O'Neill T. From "Ghettoization" to a field of its own: A comprehensive review of street harassment research. *Trauma, Violence, & Abuse* 2021:15248380211021608. doi: 10.1177/15248380211021608
5. Vera-Gray F, Kelly L. Contested gendered space: Public sexual harassment and women's work safety. In: Ceccato V, Nalla MK, eds. Crime and Fear in Public Places Towards Safe, Inclusive and Sustainable Cities. London: Routledge 2020:217-31.
6. Smith R, Gallagher M, Popkin S, et al. Coercive sexual environments: What MTO tells us about neighborhoods and sexual safety. *CityScape* 2014;16(1):85-112.
7. UN Women. Safe cities and safe public spaces: Global results report, 2017.
8. Gardner CB. Passing By: Gender and Public Harassment. Berkeley: University of California Press 1995:4.
9. Sifuna DN. A review of major obstacles to women's participation in higher education in Kenya. *Research in Post-Compulsory Education* 2006;11(1):85-105. doi: 10.1080/13596740500507995
10. Littleton H, Abrahams N, Bergman M, et al. Sexual assault, sexual abuse, and harassment: Understanding the mental health impact and providing care for survivors: An International Society for Traumatic Stress Studies Briefing Paper: International Society for Traumatic Stress Studies, 2018.
11. Gardner N, Cui J, Coiacetto E. Harassment on public transport and its impacts on women's travel behaviour. *Australian Planner* 2017;54(1):8-15. doi: 10.1080/07293682.2017.1299189
12. Eom E, Restaino S, Perkins AM, et al. Sexual harassment in middle and high school children and effects on physical and mental health. *Clinical Pediatrics* 2015;54(5):430-38. doi: 10.1177/0009922814553430
13. Houle JN, Staff J, Mortimer JT, et al. The impact of sexual harassment on depressive symtoms during the early occupational career. *Soc Ment Health* 2011;1(2):89-105. doi: 10.1177/2156869311416827
14. Chiodo D, Wolfe DA, Crooks C, et al. Impact of sexual harassment victimization by peers on subsequent adolescent victimization and adjustment: A longitudinal study. *Journal of Adolescent Health* 2009;45(3):246-52. doi: https://doi.org/10.1016/j.jadohealth.2009.01.006
15. Muasya J. Effects of sexual harassment on women students' access to opportunities and facilities: A case study of the University of Nairobi, Kenya. *Global Journal of Interdisciplinary Social Sciences* 2014;3(4):83-90.
16. United Nations. Prohibition of discrimination, harassment, including sexual harassment, and abuse of authority Secretary-General's Bulletin, 2008.
17. Abuya BA, Onsomu EO, Moore D. Educational challenges and diminishing family safety net faced by high-school girls in a slum residence, Nairobi, Kenya. *International Journal of*

For peer review only - http://bmjopen.bmj.com/site/about/guidelines.xhtml

Educational Development 2012;32(1):81-91. doi: https://doi.org/10.1016/j.ijedudev.2011.02.012
18. Loukaitou-Sideris A. Fear and safety in transit environments from the women's perspective. *Security Journal* 2014;27(2):242-56. doi: 10.1057/sj.2014.9
19. Mellgren C, Andersson M, Ivert A-K. "It Happens All the Time": Women's experiences and normalization of sexual harassment in public space. *Women & Criminal Justice* 2018;28(4):262-81. doi: 10.1080/08974454.2017.1372328
20. Bourgault S, Peterman A, O'Donnell M. Violence against women and children during COVID-19—one year on and 100 papers in. Violence Against Women and Children During COVID-19: Research Round Up. Washington, DC: Center for Global Development, 2021.
21. UN Women. Measuring the shadow pandemic: Violence against women during COVID-19, 2021.
22. World Health Organization. Health in 2015: from MDGs, millennium development goals to SDGs, sustainable development goals, 2015.
23. International Centre for Reproductive Health-Kenya (ICRHK) & PMA Agile. Nairobi Youth Respondent-Driven Sampling Survey: Final Report. 2020. Performance Monitoring for Action Technical Report. Baltimore, MD, USA: Bill & Melinda Gates Institute for Population and Reproductive Health, Johns Hopkins University Bloomberg School of Public Health, 2020.
24. Kenyatta U. Address to the Nation by h.e. Uhuru Kenyatta, c.g.h, President of the Republic of Kenya and Commander-in-Chief of the Defence Forces on COVID-19, commonly known as coronavirus at Harambee House, Nairobi on 15th March 2020 [Speech], 2020.
25. World Health Organization. Ethical and safety recommendations for intervention research on violence against women: building on lessons from the WHO publication putting women first: ethical and safety recommendations for research on domestic violence against women. Geneva, Switzerland: WHO, 2016
26. Center on Gender Equity and Health. Evidence-based Measures of Empowerment for Research on Gender Equality (EMERGE), 2017.
27. Operario D, Adler NE, Williams DR. Subjective social status: reliability and predictive utility for global health. *Psychology & Health* 2004;19(2):237-46. doi: 10.1080/08870440310001638098
28. Decker MR, Bevilacqua K, Wood SN, et al. Gender-based violence during COVID-19 among adolescent girls and young women in Nairobi, Kenya: a mixed-methods prospective study over 18 months. *BMJ global health* 2022;7(2):e007807.
29. Kenya National Bureau of Statistics, Kenya Ministry of Health, Kenya National AIDS Control Council, et al. Kenya Demographic and Health Survey 2014. Rockville, MD, USA, 2015.
30. Ministry of Labour and Social Protection of Kenya Department of Children's Services. Violence against Children in Kenya: Findings from a National Survey, 2019. Nairobi, Kenya, 2019.
31. Bhattacharjee P, Ma H, Musyoki H, et al. Prevalence and patterns of gender-based violence across adolescent girls and young women in Mombasa, Kenya. *BMC Women's Health* 2020;20(1):229. doi: 10.1186/s12905-020-01081-8
32. Wada O, Olawade D, Amusa A, et al. Gender-based violence during COVID-19 lockdown: case study of a community in Lagos, Nigeria. *African Health Sciences* 2022;2(22):79-87.

For peer review only - http://bmjopen.bmj.com/site/about/guidelines.xhtml

33. Kaltiala-Heino R, Fröjd S, Marttunen M. Sexual harassment victimization in adolescence: Associations with family background. *Child Abuse & Neglect* 2016;56:11-19. doi: https://doi.org/10.1016/j.chiabu.2016.04.005
34. Khoury-Kassabri M. Student victimization by educational staff in Israel. *Child Abuse & Neglect* 2006;30(6):691-707. doi: https://doi.org/10.1016/j.chiabu.2005.12.003
35. Khoury-Kassabri M, Benbenishty R, Avi Astor R, et al. The contributions of community, family, and school variables to student victimization. *American Journal of Community Psychology* 2004;34(3):187-204. doi: 10.1007/s10464-004-7414-4
36. Popkin SJ, Acs G, Smith R. Understanding how place matters for kids *Community Investments* 2010;22:23-26.
37. Koi VO, Auka J, Kilaha S. Perceived magnitude of sexual harassment in learning institutions: A case study of Kenya Medical Traning College, Nairobi. *International Academic Journal of Social Sciences and Education* 2018;2(1):65-73.
38. Fawole OI, Balogun OD, Olaleye O. Experience of gender-based violence to students in public and private secondary schools in Ilorin, Nigeria. *Ghana Med J* 2018;52(2):66-73. doi: 10.4314/gmj.v52i2.1 [published Online First: 2019/01/22]
39. Iliyasu Z, Abubakar IS, Aliyu MH, et al. Prevalence and Correlates of Gender-based Violence among Female University Students in Northern Nigeria. *African Journal of Reproductive Health / La Revue Africaine de la Santé Reproductive* 2011;15(3):111-19.
40. The National Strategic Framework for Violence Free Basic Education in Nigeria. Nigeria: Federal Ministry of Education, 2007.
41. Kibunja BK, Musembi HM, Kimani RW, et al. Prevalence and effect of workplace violence against emergency nurses at a tertiary hospital in Kenya: A cross-sectional study. *Safety and Health at Work* 2021;12(2):249-54. doi: https://doi.org/10.1016/j.shaw.2021.01.005
42. Blumell LE, Mulupi D. “Newsrooms need the metoo movement.” Sexism and the press in Kenya, South Africa, and Nigeria. *Feminist Media Studies* 2021;21(4):639-56. doi: 10.1080/14680777.2020.1788111
43. Quaife M, van Zandvoort K, Gimma A, et al. The impact of COVID-19 control measures on social contacts and transmission in Kenyan informal settlements. *BMC Medicine* 2020;18(1):316. doi: 10.1186/s12916-020-01779-4
44. Piquero AR, Jennings WG, Jemison E, et al. Domestic violence during the COVID-19 pandemic - Evidence from a systematic review and meta-analysis. *Journal of Criminal Justice* 2021;74:101806. doi: https://doi.org/10.1016/j.jcrimjus.2021.101806
45. Pinchoff J, Austrian K, Rajshekhar N, et al. Gendered economic, social and health effects of the COVID-19 pandemic and mitigation policies in Kenya: evidence from a prospective cohort survey in Nairobi informal settlements. *BMJ Open* 2021;11(3):e042749. doi: 10.1136/bmjopen-2020-042749 [published Online First: 2021/03/05]
46. Roesch E, Amin A, Gupta J, et al. Violence against women during covid-19 pandemic restrictions. *BMJ* 2020;369:m1712. doi: 10.1136/bmj.m1712 [published Online First: 2020/05/10]
47. The Sexual Offenses Act. In: Kenya Po, ed. No 3 of 2006. Nairobi, Kenya: Government of Kenya, 2006.
48. The Sexual Offenses Act Revised Edition. In: Kenya AGo, ed. No 3 of 2006. Nairobi, Kenya: National Council for Law Reporting, 2009.
49. Dozens protest gender-based violence in Kenya. *La Prensa* 2022.

For peer review only - http://bmjopen.bmj.com/site/about/guidelines.xhtml

50. Rabin RF, Jennings JM, Campbell JC, et al. Intimate partner violence screening tools: a systematic review. *Am J Prev Med* 2009;36(5):439-45 e4. doi: 10.1016/j.amepre.2009.01.024 [published Online First: 2009/04/14]
51. Bhalla N. Kenya orders probe into rise in violence against women and girls during pandemic. *Reuters* 2020.
52. Bhalla N. Kenya president pledges millions to fight surge in gender violence. *Reuters* 2020.
53. UN Women Africa. Kenya's platform for action revealed ahead of Generation Equality Forum, 2021.
54. Republic of Kenya. Generation equality forum: Kenya’s roadmap for advancing gender equality and ending all forms of gender based violence and female genital mutilation by 2026. Kenya: UN Women, International Center for Research on Women, United Nations Population Fund, 2021.

For peer review only

Figure 1. Trajectories of sexual harassment relative to and changes in frequency since the COVID-10 pandemic among adolescent girls and young women in Nairobi, Kenya in mid-2020 (n=612)

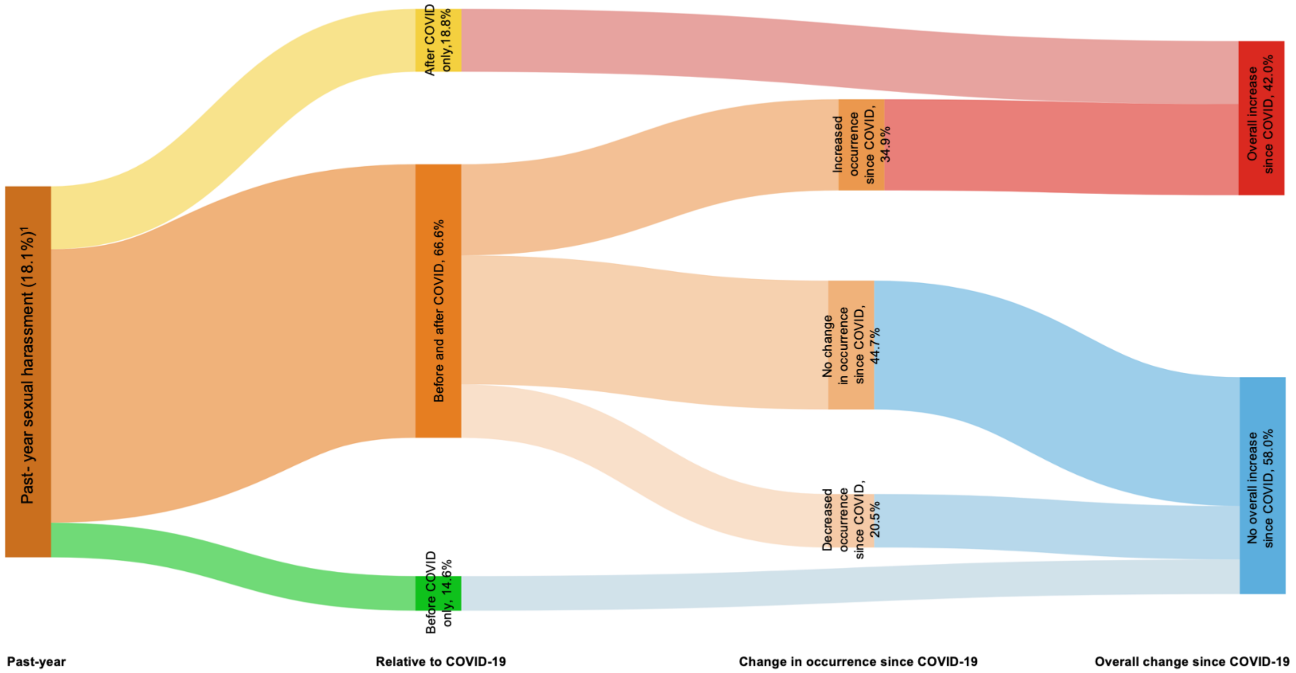

1. Among women reporting past-year sexual harassment in mid-2020

review only

STROBE Statement—Checklist of items that should be included in reports of ***cohort studies***

	Item No	Recommendation	Page No
**Title and abstract**	1	(*a*) Indicate the study's design with a commonly used term in the title or the abstract	1, 2
		(*b*) Provide in the abstract an informative and balanced summary of what was done and what was found	2
**Introduction**			
Background/rationale	2	Explain the scientific background and rationale for the investigation being reported	4-5
Objectives	3	State specific objectives, including any prespecified hypotheses	5
**Methods**			
Study design	4	Present key elements of study design early in the paper	5
Setting	5	Describe the setting, locations, and relevant dates, including periods of recruitment, exposure, follow-up, and data collection	5-6
Participants	6	(*a*) Give the eligibility criteria, and the sources and methods of selection of participants. Describe methods of follow-up	5
		(*b*) For matched studies, give matching criteria and number of exposed and unexposed	N/A
Variables	7	Clearly define all outcomes, exposures, predictors, potential confounders, and effect modifiers. Give diagnostic criteria, if applicable	6-7
Data sources/ measurement	8*	For each variable of interest, give sources of data and details of methods of assessment (measurement). Describe comparability of assessment methods if there is more than one group	6-7
Bias	9	Describe any efforts to address potential sources of bias	8
Study size	10	Explain how the study size was arrived at	7
Quantitative variables	11	Explain how quantitative variables were handled in the analyses. If applicable, describe which groupings were chosen and why	7
Statistical methods	12	(*a*) Describe all statistical methods, including those used to control for confounding	7-8
		(*b*) Describe any methods used to examine subgroups and interactions	N/A
		(*c*) Explain how missing data were addressed	N/A
		(*d*) If applicable, explain how loss to follow-up was addressed	7
		(*e*) Describe any sensitivity analyses	N/A
**Results**			
Participants	13*	(a) Report numbers of individuals at each stage of study—eg numbers potentially eligible, examined for eligibility, confirmed eligible, included in the study, completing follow-up, and analysed	7
		(b) Give reasons for non-participation at each stage	N/A
		(c) Consider use of a flow diagram	N/A
Descriptive data	14*	(a) Give characteristics of study participants (eg demographic, clinical, social) and information on exposures and potential confounders	8
		(b) Indicate number of participants with missing data for each variable of interest	N/A
		(c) Summarise follow-up time (eg, average and total amount)	N/A
Outcome data	15*	Report numbers of outcome events or summary measures over time	8

For peer review only - http://bmjopen.bmj.com/site/about/guidelines.xhtml

Main results	16	(*a*) Give unadjusted estimates and, if applicable, confounder-adjusted estimates and their precision (eg, 95% confidence interval). Make clear which confounders were adjusted for and why they were included	8-11
		(*b*) Report category boundaries when continuous variables were categorized	N/A
		(*c*) If relevant, consider translating estimates of relative risk into absolute risk for a meaningful time period	N/A
Other analyses	17	Report other analyses done—eg analyses of subgroups and interactions, and sensitivity analyses	N/A
**Discussion**			
Key results	18	Summarise key results with reference to study objectives	11-12
Limitations	19	Discuss limitations of the study, taking into account sources of potential bias or imprecision. Discuss both direction and magnitude of any potential bias	13
Interpretation	20	Give a cautious overall interpretation of results considering objectives, limitations, multiplicity of analyses, results from similar studies, and other relevant evidence	11-12
Generalisability	21	Discuss the generalisability (external validity) of the study results	13
**Other information**			
Funding	22	Give the source of funding and the role of the funders for the present study and, if applicable, for the original study on which the present article is based	2 (abstract)

*Give information separately for exposed and unexposed groups.

Note: An Explanation and Elaboration article discusses each checklist item and gives methodological background and published examples of transparent reporting. The STROBE checklist is best used in conjunction with this article (freely available on the Web sites of PLoS Medicine at http://www.plosmedicine.org/, Annals of Internal Medicine at http://www.annals.org/, and Epidemiology at http://www.epidem.com/). Information on the STROBE Initiative is available at http://www.strobe-statement.org.

For peer review only
